# Arterial Calcification and Its Association With Stroke: Implication of Risk, Prognosis, Treatment Response, and Prevention

**DOI:** 10.3389/fncel.2022.845215

**Published:** 2022-05-11

**Authors:** Xiang Wang, Xinghang Chen, Zhuohui Chen, Mengqi Zhang

**Affiliations:** ^1^Department of Neurology, Xiangya Hospital, Central South University, Changsha, China; ^2^National Clinical Research Center for Geriatric Disorders, Xiangya Hospital, Central South University, Changsha, China

**Keywords:** arterial calcification, stroke, computed tomography, radiography, calcification scoring method

## Abstract

Stroke is a leading cause of death worldwide. Vascular calcification (VC), defined as deposition of calcium-phosphate complexes in the vessels, is considered as the characteristic of vascular aging. Calcifications at different vessel layers have different implications. Intimal calcification is closely related to atherosclerosis and affects plaque stability, while medial calcification can cause arterial stiffening and reduce compliance. Accumulating evidence suggested that arterial calcifications, including calcifications in the intracranial artery, coronary artery, and carotid artery, are associated with the risk, prognosis, and treatment response of stroke. VC can not only serve as a marker of atherosclerosis, but cause cerebral hemodynamic impairment. In addition, calcifications in large arteries are associated with cerebral small vessel disease. In this review, we summarize the findings of recently published studies focusing on the relationship between large artery calcification and the risk, prognosis, treatment response, and prevention of stroke and also discuss possible mechanisms behind those associations.

## Introduction

Vascular calcification (VC) is one of the characteristics of vascular aging (Tesauro et al., 2017) and appears to specifically occur in arteries (Wu et al., 2016). It is defined as the deposition of calcium-phosphate complexes in the vessels. Apart from aging, pathological processes like diabetes mellitus, chronic kidney disease, and hereditary disorders might also be the risk factors for arterial calcification (Wu et al., 2013). Calcification at different locations in the arterial wall might be associated with different risk factors (Vos et al., 2018) and outcomes. Intimal calcification is closely related to atherosclerosis and affects the stability of the plaque, while medial calcification, including calcification located in the tunica media and around the internal elastic lamina, is considered to cause arterial stiffening and reduce compliance (Kockelkoren et al., 2017).

Vascular calcification is considered to be a complex and regulated process which has similar mechanisms in skeletal bone calcification. According to different pathologies, VC can be generally classified into three types, namely inflammatory, metabolic, and genetic (Demer and Tintut, 2014). Inflammatory type is associated with atherosclerosis and mainly takes place in the intima, which is analogous to endochondral ossification, while metabolic type mainly happens in the media and is associated with chronic kidney disease and diabetes, which is analogous to intramembranous ossification. Genetic VC is linked with genetic disorders, such as Marfan syndrome and usually takes place in the media. Previous studies indicate that chronic inflammation plays a central role in the formation of atherosclerotic calcification (Aikawa et al., 2007; Abdelbaky et al., 2013). Other important factors in VC formation include oxidized lipids, elastin, alkaline phosphatase, matrix gamma-carboxyglutamic acid protein (MGP), transglutaminase, etc. (Demer and Tintut, 2014).

Arterial calcification can take place in various vessels, such as the femoral artery, abdominal aorta, thoracic aorta, coronary artery, carotid artery, and cerebral arteries (Wu et al., 2016). Among these, the best-studied are coronary artery calcification (CAC), intracranial artery calcification (IAC), and carotid artery calcification. CAC has been used as a predictor of coronary heart disease and recent studies have shown that CAC can also predict the risk of atherosclerotic cardiovascular disease, including stroke (Budoff et al., 2018). Similarly, IAC, especially intracranial internal carotid artery calcification (IICAC), and carotid artery calcification are also reported to be closely associated with stroke (Agacayak et al., 2020; Golüke et al., 2020). Since arterial calcification can be easily detected and assessed on unenhanced computed tomography (CT), CAC, IAC, and carotid artery calcification can be used as a powerful and non-invasive marker of diseases like stroke in clinical settings. To improve the understanding of the role of arterial calcification in stroke, this review provides an overview of recently published studies focusing on the relationship between arterial calcification and the initiation, development, prognosis, treatment response, and prevention of stroke. In this review, possible mechanisms lying behind the association of arterial calcification and stroke are also discussed.

## Intracranial Artery Calcification

### Measurements of Intracranial Artery Calcification

There are various methods available for the quantification of IAC. Although IAC can be detected on histological tests or magnetic resonance imaging (MRI), unenhanced CT remains to be the best tool for the measurement of IAC due to its accuracy, relatively low cost and simplicity. So in this part, we will focus on the quantification methods of IAC on unenhanced CT.

**FIGURE 1:**
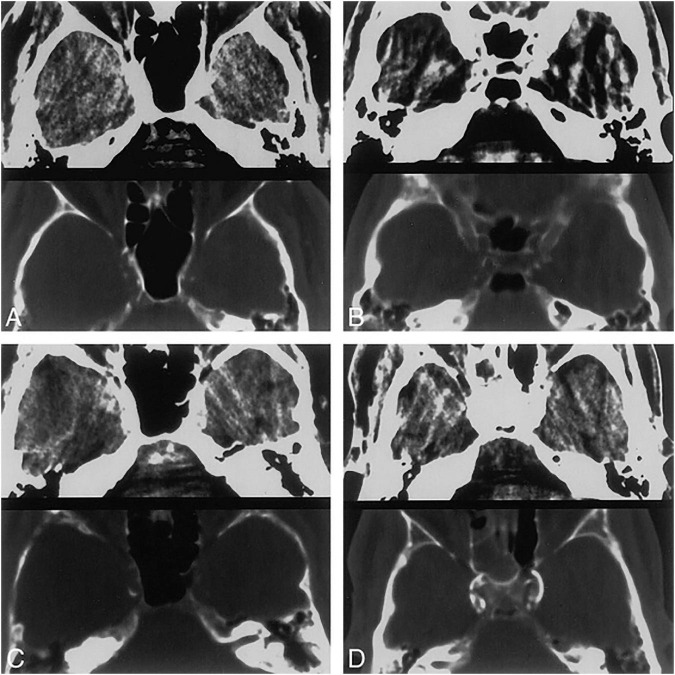
Four different CT patterns of calcification. 5-mm-thick axial CT scans at the level of the carotid siphon. Brain (top) and bone (bottom) windows are shown in each image. **(A)** Thin, discontinuous calcification, bilaterally. **(B)** Thin, continuous calcification, right side. **(C)** Thick, discontinuous calcification, right side. **(D)** Thick, continuous calcification, bilaterally. Republished with permission of American Society of Neuroradiology, from Woodcock et al. (1999); permission conveyed through Copyright Clearance Center, Inc.

Quantification methods of IAC can be generally classified into two categories, qualitative visual scores like the Woodcock visual scoring, and quantitative methods like the Agatston score and calcium volume (shown in Supplementary Table 1). In qualitative visual grading systems, the extent and thickness of calcification are assessed to give a score. The most commonly used visual grading system is the Woodcock visual scoring, where the severity of calcification is subtyped into absent, mild (thin, discontinuous), moderate (thin, continuous or thick, discontinuous), and severe (thick, continuous) (Woodcock et al., 1999) (shown in Figure 1). Other visual scoring methods include the modified Woodcock visual scoring (4-point scale) (Subedi et al., 2015), Barbiarz’s method (5-point scale) (Babiarz et al., 2003), etc. Visual grading systems are quick and simple but subjective in nature. Besides, some details in calcification might be overlooked and slight differences in calcification cannot be detected *via* visual grading systems.

In comparison, quantitative methods are more objective and can be carried out on software. The Agatston score is a semiautomatic quantitative calcium scoring that was originally used to measure CAC three decades ago. The presence of calcific lesion is determined if there are pixels at a CT density ≥ 130 HU having an area ≥ 1 mm^2^ to eliminate noise. A region of interest (ROI) is manually drawn around calcific lesions to avoid contamination from the adjacent bone. Then a score is calculated by multiplying the area and a cofactor depending on the maximal density of the plaque (1 = 130∼199 HU, 2 = 200∼299 HU, 3 = 300∼399 HU, and 4 ≥ 400 HU) (Agatston et al., 1990). In 2006, Taoka et al. (2006) utilized the Agatston score to measure IAC and found that there was a correlation between calcium score and angiographic changes of atherosclerosis in the carotid siphon and bifurcation. de Weert et al. (2009) developed another scoring method based on calcium volume. They developed a custom-made plug-in for the ImageJ software so that polygonal ROI can be manually drawn. Calcium volume is automatically calculated by multiplying the number of pixels above the threshold (normally 130 HU), the pixel size and the increment. The main drawback of quantitative methods is that ROI has to be manually drawn to exclude adjacent bone, which is the only time-consuming part.

It can be seen that measurements of IAC are diverse and inconsistent. Despite excellent correlation was found between the Woodcock visual scoring and the Agatston score or calcium volume (Subedi et al., 2015), Ahn et al. (2013a) found that visual grades poorly reflect actual calcium volume. The inconsistency in the measurement of IAC makes it difficult to compare results from different studies. Thus, a reproducible, accurate, and practical scoring method is urgently needed.

In addition to CT, new imaging techniques for detecting calcification are also emerging. For instance, susceptibility weighted imaging (SWI) could differentiate calcification from blood products and achieve a performance comparable to CT (Adams et al., 2017). In the future, radiation-free SWI might be the alternative to CT for detecting calcification.

### Intracranial Artery Calcification and Risk of Stroke

**FIGURE 2:**
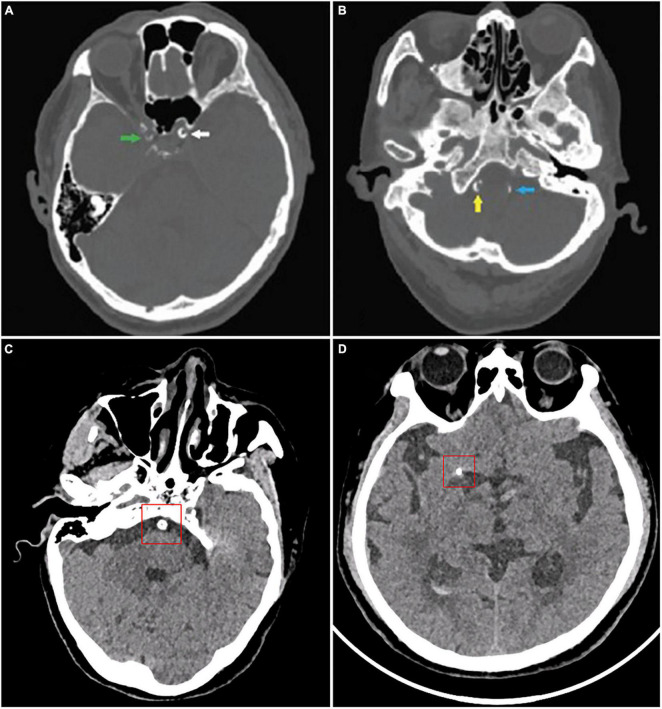
CT images of intracranial artery calcification. According to Babiarz’s method, calcifications were graded as follows: **(A)** Calcifications in the right internal carotid artery (green), 3 for extent and 2 for thickness; left internal carotid artery (white), 4 for extent and 3 for thickness. **(B)** Calcifications in the right vertebral artery (yellow), 3 for extent and 3 for thickness; left vertebral artery (blue), 2 for extent and 2 for thickness. According to Woodcock scoring, calcifications were graded as follows: **(C)** Calcifications in the basilar artery, severe (thick, continuous). **(D)** Calcifications in the middle cerebral artery calcification, moderate (thick, discontinuous). **(A,B)** Were from Wu et al. (2016).

A number of studies have looked into the association between IAC and risk of stroke in a variety of populations. According to a cohort study of 276 patients with transient ischemic attack (TIA) or acute ischemic stroke (AIS), intracranial internal carotid artery (IICA) is the most commonly affected site of calcification (incidence rate 64.8%), followed by vertebral artery (30.2%), basilar artery (19.5%), and middle cerebral artery (MCA) (6.3%) (CT images are shown in Figure 2) (Chen et al., 2019). Other studies also reported similar results (Koton et al., 2012; Quiney et al., 2017; Strobl et al., 2018).

**FIGURE 3:**
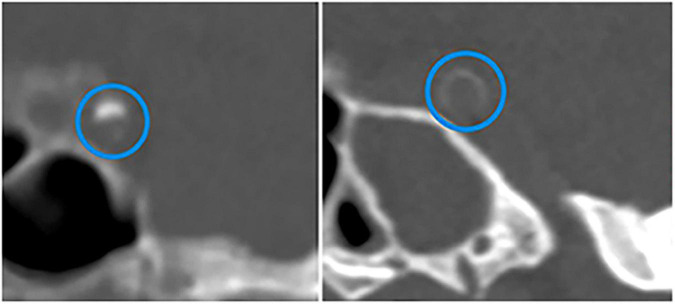
CT images of calcifications in different layers of intracranial internal carotid arteries. Intimal calcification (the left) and medial calcification (the right) are marked by blue circles. Adapted from Kockelkoren et al. (2017).

Some studies measured total calcification in the intracranial arteries, while others investigated the relationship between calcification of a certain artery (e.g., IICA, vertebral artery, MCA) and stroke. A retrospective study of 175 patients older than 60 found that the total visual score of IAC was significantly higher in subjects with the occurrence of major adverse cardiovascular events (including stroke) than those without major adverse cardiovascular events (Strobl et al., 2018). According to the Rotterdam Study involving 2,524 white participants (mean age, 69.5 years), the presence of IICAC or larger IICAC volumes were associated with a higher risk of any stroke and ischemic stroke (Bos et al., 2014). Another study of patients with ischemic stroke or TIA reported that the presence of IAC was significantly associated with increased downstream stroke/TIA (Quiney et al., 2017). Golüke et al. used a scoring model developed by Kockelkoren et al. (2017) to distinguish intimal calcification from medial calcification (Figure 3) and Woodcock visual scoring to measure IICAC. They found that intimal calcification, medial calcification, and severity of calcification in IICA were all associated with stroke (Golüke et al., 2020). Recently, a histopathology study of 211 autopsy cases also revealed that the presence of calcification in the circle of Willis was associated with ischemic infarcts (Shapiro et al., 2019). Combined together, these results substantiated an association between IAC and the risk of stroke.

According to the Trial of Org 10172 in Acute Stroke Treatment (TOAST), ischemic stroke can be classified into five subtypes: (1) large-artery atherosclerosis, (2) cardioembolism, (3) small-vessel occlusion, (4) stroke of other determined etiology, and (5) stroke of undetermined etiology (Adams et al., 1993). IAC might have different contributions to different stroke subtypes. VC, especially intimal calcification, is generally considered to be a marker of atherosclerosis (Wu et al., 2016). A postmortem study recruited 32 cases with 96 arteries analyzed and found that in IAC, intimal calcification is most common and is related with progressive atherosclerotic lesions (Yang et al., 2018). This is contradictory to a histological study involving 20 cases and 39 arteries which showed that calcifications in IICA were predominantly located around the internal elastic lamina and that this type of calcification was not related to atherosclerosis (Vos et al., 2016). More studies with larger sample sizes are needed to determine the dominant type of calcification in intracranial arteries.

Recently, a retrospective study included 1,161 patients and used a visual score to assess the calcification in the cavernous portion of IICA. They found that calcification score could reflect the overall cerebral atherosclerosis burden and the presence of calcification could be used to detect cerebral atherosclerosis with a high sensitivity of 84% (Kim et al., 2019). Yilmaz et al.’s study revealed that patients with severe IICAC were more likely to have large-artery atherosclerosis or cardioembolism as the underlying cause of stroke. Moreover, they found that the severity of IICAC was associated with atherosclerotic vascular risk factors and the presence of calcification in other vascular beds, including the aorta arch, carotid artery, extracranial vertebral artery, and other intracranial arteries (Yilmaz et al., 2015). This is in line with another study which showed that there was a weak correlation between the calcium volume of the intracranial carotid artery and vertebrobasilar artery (VBA) (van der Toorn et al., 2019). In addition, IAC is found to significantly correlate with CAC for both Agatston score and calcium volume (Ahn et al., 2013b). Taken together, the above findings might indicate that IICAC or IAC is a marker of atherosclerotic burden in the whole body. However, it is worth noting that large calcifications and small calcifications might own different pathologies and their relationships with atherosclerosis differ (Shioi and Ikari, 2018). Recently, a histopathological study categorized calcifications in the circle of Willis into large calcifications (“coalescent”), which can be detected on CT, and small calcifications (“scattered”), which are likely undetectable on CT. They found that small calcifications were associated with cholesterol-driven intracranial large artery atherosclerosis while large calcifications were not associated with atherosclerosis (Shapiro et al., 2019). Small calcification, or microcalcification, is not merely a process induced by atherosclerotic inflammation but it can also elicit a pro-inflammatory response in macrophages (Nadra et al., 2005). In contrast, large calcification, or macrocalcification, is linked with a healing process after inflammatory response (Shioi and Ikari, 2018).

Cerebral small vessel disease (SVD) is a term used to describe all the pathological processes that affect the small vessels of the brain, including small arteries, arterioles, venules, and capillaries (Pantoni, 2010). The consequences of SVD are lacunar infarcts, white matter lesions (WML) or white matter hyperintensities (WMHs), cerebral microbleeds (CMBs), enlarged perivascular spaces (EPVS), which are easily identified on brain MRI and are considered markers of SVD. SVD is also a common cause of stroke (Pantoni, 2010). Recently, a number of studies have identified the association between IAC and SVD. Chen et al. (2019) found that the severity of IAC was associated with the presence of WMHs and lacunes. Another two studies also showed that calcifications in the carotid siphon were associated with moderate-to-severe WMHs and lacunar infarcts (Hong et al., 2011; Del Brutto et al., 2016). However, when it comes to the correlation between IAC and CMBs or EPVS, research findings are rather inconsistent. In 2011, research based on the Rotterdam Study including 885 people aged 60 years or older showed that there was no association between IICAC and CMBs (Bos et al., 2011). Later, a prospective study enrolled 834 patients and used a visual score to assess IICAC. They found that IICAC was associated with a higher risk of CMBs (Chung et al., 2014). Then in 2019, Chen et al. conducted a cross-sectional study and used the Agatston score to analyze the calcification of 276 patients with AIS or TIA. They reported that IAC severity was associated with the presence of CMBs (Chen et al., 2019). The inconsistency between these studies might be due to differences in study populations, the definition of CMBs and calcification measurements. In 2017, a retrospective study enrolled 189 patients with ischemic stroke due to MCA occlusion and used the Woodcock visual score to assess calcification. They found that calcification in the carotid siphon was negatively correlated with perivascular spaces (Tao et al., 2017). In 2019, Chen et al. (2019) found no association between IAC and EPVS. In 2020, an observational study categorized 581 community-dwelling individuals into low calcium group and high calcium group by the Woodcock visual score. They found that high calcium content in the carotid siphon was independently associated with the presence of >10 EPVS in the basal ganglia (Del Brutto et al., 2020). The three studies are highly contradictory to each other and more studies are needed to figure out whether there is a relationship between IAC and EPVS.

To take a further step, the reason why calcifications in large cerebral arteries can affect SVD involving small vessels still requires extensive research. Calcification might cause arterial stiffening and thus limitation of vasodilation, which leads to chronic hypoperfusion in the small perforating artery territory resulting in parenchymal damage such as WMHs or lacunar infarcts (Chung et al., 2010). Besides, calcification might also cause arterial stenosis (Baradaran et al., 2017; Yang et al., 2018), which also leads to hypoperfusion in the small arteries (Del Brutto et al., 2016).

Intracranial artery calcification is also linked with stroke of undetermined etiology, or cryptogenic stroke. A study showed that in patients with cryptogenic stroke, the calcium burden was greater in the IICA on the affected side than that in the IICA on the unaffected side, while in patients with cardioembolic stroke as the control group, no differences were found between calcium burden in the ICA on the affected and the unaffected side (Kamel et al., 2017). This suggests that some cryptogenic stroke is in fact due to upstream artery atherosclerosis and should be classified as large-artery atherosclerotic stroke. Hence, incorporating IAC into the classification system of ischemic stroke might be promising since it can help determine some of the so-called “cryptogenic stroke” as large-artery atherosclerotic stroke, and thus is good for the treatment or prevention therapies of this subtype of stroke.

The mechanism behind the association between IAC and stroke has drawn researchers’ interest. IAC not only is a marker of atherosclerosis, but also can cause cerebral hemodynamic impairment. Wu et al. investigated the effect of IAC on cerebral hemodynamic parameters and found that heavier IAC was associated with higher MCA velocity, higher VBA velocity, and higher VBA pulsatility index, suggesting that IAC might cause generalized artery flow velocity elevation within the whole brain vasculature and arterial stiffness in VBA (Wu et al., 2018).

Although all the above mentioned studies suggest that the presence or severity of IAC is linked with a higher risk of stroke, it might be the opposite when stenosis already exists. A cross-sectional study showed that among stenotic vessel segments, IAC may decrease the risk of downstream stroke/TIA compared with non-calcified stenosis (Quiney et al., 2017). In patients with ischemic stroke who had MCA stenosis, MCA calcification was independently associated with asymptomatic MCA stenosis (stenotic MCA when there is no infarction in the ipsilateral MCA territory) (Baek et al., 2017). Another study also found that there was no significant association between a higher degree of MCA calcification and higher risk of ischemic stroke, after adjustment for the presence of MCA calcification and the confounding factors (Kao et al., 2015). Besides, a histological study involved plaques taken from patients undergoing endarterectomy of the internal carotid artery and showed that enlarged calcifications (calcifications that occupy over half of the plaques’ thickness) were independently associated with the asymptomatic characteristic of the plaques (Masztalewicz et al., 2019). An explanation is that although IAC is an indicator of atherosclerosis, calcification itself might increase the stability of the plaque (Wu et al., 2014; Jin et al., 2021), reducing the risk of plaque rupture and stroke. However, this should be interpreted cautiously since the association between calcification and plaque stability is rather complex, which depends on the size, spacing, location, and other characteristics of calcification (Maldonado et al., 2012; Hutcheson et al., 2016; Shioi and Ikari, 2018). In summary, IAC is a reliable marker of atherosclerosis and can be used to predict the risk of stroke but when there is already arterial stenosis in patients, calcification might decrease the risk of stroke by stabilizing the plaque.

### Intracranial Artery Calcification and Prognosis of Stroke

Besides being an indicator for the risk of stroke, IAC is also associated with the prognosis of stroke. Several studies have demonstrated that IAC is associated with recurrence and mortality in stroke patients. Bugnicourt et al. (2011) used a scoring method that scores the number of calcified intracranial arteries instead of the severity of calcification of each artery and found that in patients with stroke or TIA, a higher IAC score was associated with a higher risk of a major clinical event (including all-cause death, ischemic stroke, TIA, cardiovascular ischemic event, and peripheral artery event) after hospital discharge. Another study used the same scoring method to measure IAC and discovered that IAC score was an independent predictor for the recurrent event (including ischemic stroke, TIA, and ischemic heart disease) or death (Ovesen et al., 2013). Similarly, IAC Agatston score is related to a higher risk of stroke recurrence or post-stroke mortality (Wu et al., 2020)and IAC visual score is associated with an increased risk of early progression/recurrence and poor functional outcomes (Koton et al., 2012; Lee et al., 2014). In patients with TIA, a higher calcium volume was associated with a higher risk of recurrent TIA or AIS (Kong et al., 2019). In addition, two studies found that in patients with AIS, the presence of VBA calcification is also associated with long-term risk of recurrent stroke (Gökçal et al., 2018)and mortality (Magdič et al., 2020). As for stroke subtypes, IAC was associated with a higher risk of recurrence only in patients with index small-vessel occlusive stroke (Wu et al., 2020), suggesting that IAC might serve as a valuable predictor of recurrence in this subtype of stroke.

### Intracranial Artery Calcification and Response to Revascularization Treatment

Treatment of ischemic stroke includes intravenous thrombolysis (IVT) and endovascular thrombectomy, which both reduce disability (Campbell et al., 2019). However, intravenous thrombolysis is accompanied by an approximate 5.6% risk of symptomatic intracranial hemorrhage (sICH), a severe complication that is associated with high mortality (Seet and Rabinstein, 2012). Recent studies have shown that IAC can probably affect the prognosis of ischemic stroke patients after these revascularization treatments. In 2015, a study enrolled 80 patients who received IVT or endovascular thrombectomy for MCA trunk occlusion and categorized them into the low or high calcification burden (LCB or HCB) group according to IAC scores determined by the number of calcified intracranial arteries. They found that compared with the LCB group, the HCB group exhibited poorer outcomes after treatment. In this study, treatment outcome was measured by National Institutes of Health Stroke Scale (NIHSS) score indicating the severity of stroke and the modified Rankin Scale (mRS) score indicating functional dependence of patients (Lee et al., 2015).

Four studies have investigated the relationship between IAC and the prognosis of patients undergoing endovascular thrombectomy. In 2014, a retrospective study of 117 patients showed that IICAC did not influence recanalization or mRS score after endovascular thrombectomy (Haussen et al., 2014). In 2017, an observational study used the calcium volume to measure IICAC in 285 patients with AIS of the anterior circulation treated with mechanical thrombectomy. They found that both larger IICAC volume in the symptomatic carotid artery and larger total IICAC volume were associated with worse post-procedural revascularization and poorer functional outcome (Hernández-Pérez et al., 2017). However, another randomized controlled study assessed the IICAC volume of 344 AIS patients and found that IICAC volumes were not associated with functional outcome, final recanalization status, and final infarct volume (Compagne et al., 2018). The discrepancy between the two studies might be due to the differences in selection of patients and study design. In the former study, patients were only eligible for endovascular thrombectomy if they had contraindication for intravenous thrombolysis and specific clinical and imaging criteria (NIHSS score ≥ 6, etc.) were met. In contrast, the patients included in the latter study could more reflect the general population encountered in clinical practice. The randomized controlled design of the latter study might also be better. In 2020, a study enrolled 64 patients who underwent endovascular thrombectomy for posterior circulation large vessel occlusion and explored the relationship between VBA calcification (VBAC) and outcome after therapy. They found that both the presence of VBAC and larger VBAC volume were associated with reduced functional independence (mRS score 0–2) and increased 7-day and 90-day mortality (Diprose et al., 2020).

When it comes to the effect of IAC on the prognosis after IVT, research findings are inconsistent. In 2013, a retrospective and multicenter study by Lin et al. classified 297 patients into two groups (no to mild IICAC and moderate to severe IICAC) based on Woodcock visual score and found that moderate to severe IICAC were independent predictors of ICH after IVT. In addition, they found that moderate to severe IICAC increased the odds of functional dependency (mRS score 3–6) at 1 month and 1 year, though this association was confounded by other stroke risk factors (Lin et al., 2013). In 2018, a unicenter study included 448 AIS patients undergoing IVT and used Barbiarz’s method to assess total carotid siphon calcification (TCSC). They found that TCSC was an independent predictor of mortality but it did not predict sICH, recanalization or good functional outcome (mRS score 0–2) (Tábuas-Pereira et al., 2018). Later, a study enrolled 232 patients with AIS and performed an assessment of IICAC through modified Woodcock scale. No associations were found between IICAC score and sICH, functional outcome and mortality after IVT in AIS patients (He et al., 2019). In 2020, a retrospective study included 242 patients and found that IAC volume on the lesion side was associated with ICH and that the presence of IAC and the number of calcified intracranial arteries were associated with poor prognosis (mRS score > 2) (Yu et al., 2020). Overall, the results are highly controversial, which is likely due to the inconsistency of calcification measurements, inclusion and exclusion criteria and definition of sICH. Besides, evaluating the whole calcification range allows slight differences in calcification to be captured and might outperform dichotomized grouping methods used in the study by Lin et al. (2013). Further studies with larger sample sizes and better designs are needed to clarify the relationship between IAC and prognosis after IVT, including the occurrence of sICH, functional outcome, recanalization rate, and mortality.

Calcification in different layers of the artery might have different impacts on the prognosis after revascularization treatments. Therefore, several studies classified calcification into intimal and medial calcification using a previously described scoring method (Kockelkoren et al., 2017) and investigated the effect of different calcification patterns on the prognosis after treatment. In 2018, a MR CLEAN group analysis was conducted and 344 AIS patients undergoing endovascular thrombectomy or no treatment were included. A significant treatment effect, measured by mRS score, was only observed in patients with medial IICAC but not in patients with intimal IICAC (Compagne et al., 2018). Then in 2021, a perspective and multicenter study also reported similar findings. They enrolled 982 patients undergoing IVT or no treatment and found that the beneficial effect of IVT on the 90-day mRS was significant in the group with medial IICAC and in the group without IICAC, but not in the group with intimal IICAC. They also found that IVT was significantly associated with recanalization only in the group with medial IICAC and a medial IICAC pattern was significantly associated with good collateral status. Besides, no significant associations were observed between IVT and ICH in any IICAC groups (Kauw et al., 2021). These two studies have shown that the treatment effect of IVT or endovascular thrombectomy is better in patients with medial calcification rather than intimal calcification. Medial calcification leads to arterial stiffening and high pulse pressures, which could impair the distal cerebral microcirculation and then trigger the formation of collateral vessels (Kauw et al., 2021). Nonetheless, a former study reported that the presence of medial IICAC was negatively linked with early dramatic response to IVT and the frequency of sICH was higher in patients with medial calcification (Gocmen et al., 2018). However, the study only included cardioembolic and cryptogenic stroke patients with a relatively small sample size (*n* = 91) and the two associations observed were only marginally significant.

In summary, IAC can not only serve as a predictor of stroke recurrence and mortality, but also has a probable effect on the prognosis after revascularization therapies, including sICH, recanalization rate, functional outcome, and post-intervention mortality.

## Coronary Artery Calcification

### Measurements of Coronary Artery Calcification

The most classic and commonly used scoring method of CAC is the Agatston score, which has been mentioned above. In 1991, Janowitz et al. (1991) described a method for assessing the magnitude of CAC based on a single score that quantified the extent and density of computed tomography CAC, which became known as the Agatston score. Clinical risk increases with each increment of CAC score, as consistently demonstrated in numerous studies.

**FIGURE 4:**
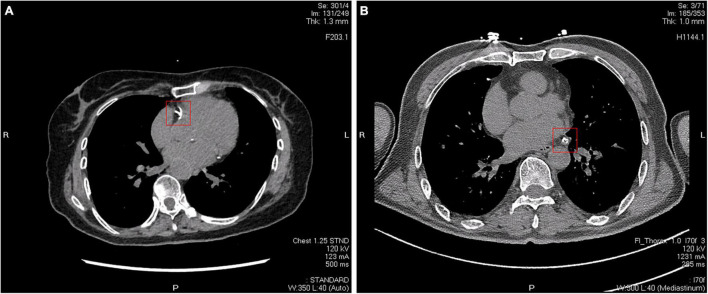
Two different CT images of coronary artery calcification. **(A)** Calcifications in the right coronary artery indicated by a red square. **(B)** Calcifications in the circumflex branch of the left coronary artery indicated by a red square.

Recently, studies have begun to address what additional information can be derived from CAC scanning (radiography images are shown in Figure 4). Budoff et al. (2007) and Tota-Maharaj et al. (2015a,b) demonstrated that the number of vessels with CAC score > 100, location of CAC, and the number of calcific lesions were all predictors of coronary events. In another study, Criqui et al. (2014) found that CAC density was inversely related to cardiovascular disease (CVD) events for a given CAC volume and was more predictive than the CAC score. This observation supports the concept that denser plaques are likely to be older healed plaques, whereas less dense plaques may be more likely to represent newer, more active plaques, with higher lipid content, and greater likelihood of inflammation (Motoyama et al., 2007, 2015).

However, there are also several factors that have a huge impact. The spherical shape and pericardial-sided location of CAC are associated with fewer CVD events and may represent morphological features related to stable coronary plaques (Foldyna et al., 2019). High calcified plaque volume (CPV) was associated with incident major adverse cardiac events (Jin et al., 2021), which is not included in the Agaston score. The role of CAC density, distribution, morphology, and location should be considered when evaluating current CAC scoring systems. Although there are studies trying to add more properties to the Agaston score (Blaha et al., 2016), we might need a new scoring method that can fully include the various properties of CAC.

In 2018, Khera et al. (2018) developed a mobile application and web-based tool to facilitate clinical application of the Astro-CHARM tool, which is the first integrated atherosclerotic cardiovascular disease (ASCVD) risk calculator to incorporate risk factors including CAC.

### Coronary Artery Calcification and Risk of Stroke

Some studies have tracked the association between the severity of CAC and the increased risk of stroke over a long period of time. The Multi-Ethnic Study of Atherosclerosis (MESA) showed that the presence of CAC was linked with lower cerebrovascular event-free survival and that ln (CAC + 1) was an independent predictor for stroke in both univariate and multivariable models. Incorporating CAC into the Framingham stroke risk score could improve the discrimination for cerebrovascular events (Gibson et al., 2014). In 2020, research showed that atrial fibrillation (AF) patients with incidental CAC depicted on chest CT had an increased risk of stroke and mortality beyond established risk factors (Hillerson et al., 2020), which is also supported by a recent study (Wang et al., 2021). In a study combining two large cohorts, a high CAC burden was predictive of stroke risk in the overall cohort and Blacks (Mehta et al., 2020). Ten-year ASCVD event rates increased steadily across CAC categories regardless of age, sex, or race/ethnicity. For each doubling of CAC, there is a 14% relative increment in ASCVD risk, holding all other risk factors constant (Budoff et al., 2018). In addition, patients whose CAC Agatston scores were greater than 1,000 had a serious risk of stroke (Rosenblit, 2019).

Subclinical cerebrovascular diseases (SCVDs) play a vital pathogenetic role in stroke (Oh et al., 2015). In 2020, a study (Khan et al., 2020) evaluated the clinical relationship between CAC and SCVDs in a healthy Japanese male population. The research used Agatston method to score CAC and categorized the participants into no CAC (0), mild CAC (1–100), and moderate-to-severe CAC (>100). Logistic regression was used to calculate the adjusted odds ratio for universal SCVDS with CAC free group as the reference. The results showed significant trends that higher CAC scores were associated with a higher incidence of the following SCVDs: lacunar infarction, deep, and subcortical white matter hyperintensity, and intracranial arterial stenosis. It directly proved that CAC and SCVDs coexist. The presence and degree of CAC may be a useful indicator for SCVDs involving small and large vessels. The coexistence of CAC and SCVDs provides a mechanism that may be used to predict stroke in the early stage.

From 1997 to 2009, a total of 23,637 participants without ASCVD underwent CAC scoring. Results were assessed using Cox proportional risk models, controlling for baseline risk factors, atrial fibrillation, and competitive mortality. The results showed that CAC scores were positively related with the risk of stroke. CAC scores significantly improved the accuracy of long-term prognosis for major adverse cardiovascular events and mortality, regardless of age and risk factors. These results support CAC screening to improve individual ASCVD risk assessment and prevention in low-risk youth (Mitchell et al., 2018b).

### Coronary Artery Calcification and Prevention of Stroke

Aside from being an indicator for the risk of stroke, CAC can also play a role in the prevention of ischemic stroke. In 2018, a study tracked a total of 4,720 individuals from the Multi-Ethnic Study of Atherosclerosis to evaluate those in most need of statin therapy to reduce ischemic stroke/transient ischemic attack risk (TIA), with a medium follow-up of 13.1 years (Osawa et al., 2018). All participants were 45–84 years old and had no known clinical CVD at the time of enrollment. In addition to traditional risk factors, the researchers used the Cox proportional hazards models to examine CAC and carotid artery intima-media thickness (CIMT) as predictors of ischemic stroke/TIA. After adjustment for traditional risk factors, the risk of stroke/TIA increased progressively with each CAC category (0, 1–100, >100) in individuals with CIMT > 75%, compared with those without CAC and with CIMT ≤ 75%. The results showed that a combination of CAC and CIMT information is promising for more accurate risk assessment of ischemic stroke/TIA, and this approach may identify individuals at low or high risk of ischemic stroke/TIA for statin therapy in primary prevention.

In 2018, a study (Mitchell et al., 2018a) identified consecutive subjects without pre-existing ASCVD or malignancy who underwent CAC scoring from 2002 to 2009 at Walter Reed Army Medical Center. A total of 13,644 patients (mean age 50 years; 71% men) were followed for a median of 9.4 years. The results showed that the effect of statin use on major adverse cardiovascular event (MACE) was significantly related to the severity of CAC. Therefore, the presence and severity of CAC identified patients without baseline ASCVD who are most likely to benefit from statins for the primary prevention of cardiovascular diseases.

In addition to statin, aspirin can be used for primary prevention of CVD, although with an increased risk of bleeding. Recent studies suggested that CAC score might also aid in selecting suitable patients that benefit from aspirin therapy. Cainzos-Achirica et al. included 6,470 participants from the Multi-Ethnic Study of Atherosclerosis (MESA) and proved that CAC might outperform the Pooled Cohort Equations (PCE) in guiding allocation of aspirin in primary prevention. The study showed that a CAC score of at least 100 could identify patients most likely to gain a net benefit from aspirin therapy, while those with CAC score = 0 should avoid the use of aspirin for prevention (Cainzos-Achirica et al., 2020). Likewise, Ajufo et al. (2021) included 2,191 participants from the Dallas Heart Study and observed a net benefit in patients with CAC of at least 100, but only in the setting of lower bleeding risk and PCE > 5%.

In conclusion, CAC can be used as an important indicator of stroke, and the severity of CAC determines whether the use of statins or aspirin to prevent future stroke can be effective.

## Carotid Artery Calcification

**FIGURE 5:**
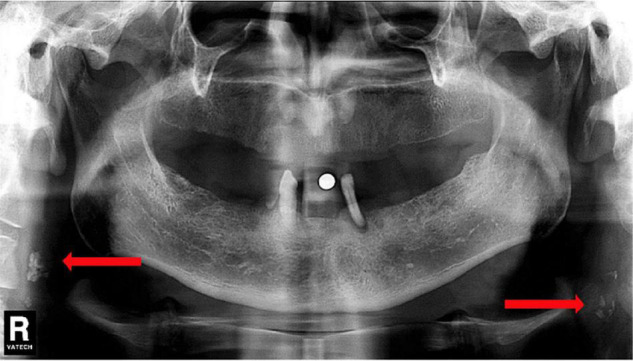
Dental Pantomogram (DPT) in which arrows indicate carotid artery calcifications. Reproduced from Ribeiro et al. (2018). Copyright© 2018 Elsevier Masson SAS. All rights reserved.

Although the association between intracranial cervical calcification and stroke has been well-proved in many studies, the extracranial segment of the carotid artery has received lesser attention. Carotid artery calcification can be seen on panoramic dental radiographs (Friedlander, 2013), which is usually located posterior to and above the mandibular angle, approximately at the lower edge of the third cervical spine near the hyoid bone (Friedlander and Baker, 1994) (shown in Figure 5). According to the identification method of carotid artery calcification that Friedlander and Lande proposed, at C3–C4 disk level or below the posterior mandibular area, about 10 lumps of radial colliculate mass adjacent to the cervical spine, which are not attached to the hyoid and usually at an angle of 45° from the mandible angle, are considered as carotid artery calcification (Friedlander and Lande, 1981).

Studies have shown that carotid artery calcification is associated with a history or future diagnostic events of stroke (Christou et al., 2010; Johansson et al., 2015). In 2019, a study searched for evidence of carotid calcifications from individuals (60–96 years) with no previous history of stroke and/or ischemic heart diseases. The data showed a significant association between carotid calcification and a future event of stroke and/or ischemic heart diseases. And the association was more pronounced in the younger group (60–72 years). The results still persisted after adjusting for other factors (hypertension, type 2 diabetes, and BMI). Also, individuals with positive carotid artery calcification had a lower mean cumulative stroke survival time (Bengtsson et al., 2019). This indicates that carotid artery calcification can increase the risk of stroke. The occurrence of carotid artery calcification was significantly correlated with the history of cerebral infarction (Kumagai et al., 2007). A study in 2020 also came to the conclusion that accidental detection of carotid artery calcification in standard panoramic radiographs for dental patients may be an important marker for preventing serious risks such as coronary artery disease, stroke, and death (Agacayak et al., 2020). Therefore, more attention should be paid to the lateral area during examination (Baumann-Bhalla et al., 2012). Multislice spiral CT can also be used to assess the relationship between carotid artery aortic arch and coronary artery calcification and stroke. A total of 2,521 persons (mean age 69.7 ± 6.8 years, 48% male) were included in the Rotterdam study. Results showed that there was a strong hierarchical association between prevalence of stroke and carotid artery calcification [OR quartile 4 vs. 1 (95% CI): 5.0 (2.2–11.0)], independent of cardiovascular risk factors. And after additional adjustment for calcification in other vascular beds, common stroke was still significantly associated with carotid artery calcification (Elias-Smale et al., 2010).

In summary, carotid artery calcification is significantly associated with a future event of stroke, which may serve as an indicator to assess the risk of stroke.

## Conclusion

**FIGURE 6:**
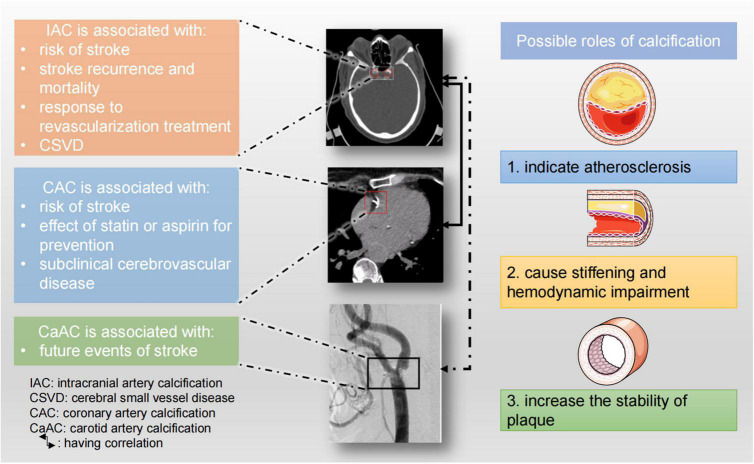
Clinical implications of large artery calcifications and possible roles of vascular calcification. The CT image of IAC was from Rahmani et al. (2022). The CT angiogram of carotid artery calcification was reused with permission of American Society of Neuroradiology, from Tsutsumi et al. (2008); permission conveyed through Copyright Clearance Center, Inc.

In summary, arterial calcification including IAC, CAC, and carotid calcification can predict the risk of stroke and it also affects treatment response and prognosis of stroke patients. Arterial calcifications and stroke share many risk factors, and in fact, history of ischemic stroke is one of the risk factors for IAC. Arterial calcification might affect plaque stability or cause hemodynamic changes, and therefore increase the risk of stroke. Besides, IAC or CAC indicates atherosclerosis, which is one of the main causes of stroke (Figure 6). It is worth noting that besides the total amount of calcification, the morphology, distribution, and size (large or small) of calcification can also impact on the risk of stroke. Intimal calcification and medial calcification have distinct implications and should be studied separately.

Previously published studies are quite inconsistent, which might be due to different study populations and different scoring methods. More studies involving large populations are still needed to resolve those discrepancies. Current calcification scoring methods have their limits and a more comprehensive, accurate, and practical scoring method is urgently needed. In the future, more advanced detection techniques, like SWI, might replace CT, and accelerate the clinical use of arterial calcification.

## Ethics Statement

This study was approved by the Ethics Committee of Xiangya Hospital of Central South University. Written informed consent was obtained from the patients in this study.

## Author Contributions

XW and XC: writing–original draft preparation. ZC: conceptualization, supervision, and writing–review and editing. MZ: conceptualization, supervision, funding acquisition, and validation. All authors have read and agreed to the published version of the manuscript.

## Conflict of Interest

The authors declare that the research was conducted in the absence of any commercial or financial relationships that could be construed as a potential conflict of interest.

## Publisher’s Note

All claims expressed in this article are solely those of the authors and do not necessarily represent those of their affiliated organizations, or those of the publisher, the editors and the reviewers. Any product that may be evaluated in this article, or claim that may be made by its manufacturer, is not guaranteed or endorsed by the publisher.
