# Supplementary material for: Arterial Calcification and Its Association With Stroke: Implication of Risk, Prognosis, Treatment Response, and Prevention
Source: Front Cell Neurosci. 2022 May 11;16:845215. doi: 10.3389/fncel.2022.845215 (PMC9130460; doi:10.3389/fncel.2022.845215)
Supplement: Supplementary file 1 [file Table_1.DOCX]

Supplementary Material

**Supplementary Table 1** Different scoring methods of IAC.

| **Method** | **Description** |
| --- | --- |
| **Visual scoring methods:** | |
| Woodcock visual scoring | |
| absent | No calcification |
| mild | Thin, discontinuous |
| moderate | Thin, continuous or thick, discontinuous |
| severe | Thick, continuous |
| Barbiarz’s method | |
| Extent of calcification | |
| Grade 0 | No calcification |
| 1 | Dot of calcification |
| 2 | Crescentic area of calcification involving <90 degrees of wall circumference |
| 3 | Calcification involving 90~270 degrees of wall circumference |
| 4 | Calcification involving 270~360 degrees of wall circumference |
| Thickness of calcification | |
| Grade 0 | No calcification |
| 1 | Calcification 1 mm thick |
| 2 | Calcification 2 mm thick |
| 3 | Calcification 3 mm thick |
| 4 | Calcification >3 mm thick |
| **Quantitative scoring methods:** | |
| The Agatston score | A score is calculated by multiplying the area and a cofactor depending on the maximal density of the plaque (1=130~199 HU, 2=200~299 HU, 3=300~399 HU and 4 ≥400 HU) after the threshold for calcification is set and ROI is manually drawn. |
| Calcium volume | Calcium volume is calculated by multiplying the number of pixels above the threshold (normally 130 HU), the pixel size and the increment after ROI is manually drawn. |
